# An OFDM Receiver with Frequency Domain Diversity Combined Impulsive Noise Canceller for Underwater Network

**DOI:** 10.1155/2015/841750

**Published:** 2015-08-17

**Authors:** Rie Saotome, Tran Minh Hai, Yasuto Matsuda, Taisaku Suzuki, Tomohisa Wada

**Affiliations:** ^1^Department of Information Engineering, University of the Ryukyus, 1 Senbaru, Nishihara, Okinawa 903-0213, Japan; ^2^Graduate School of Engineering and Science, University of the Ryukyus, 1 Senbaru, Nishihara, Okinawa 903-0213, Japan; ^3^Department of Media Information Engineering, Okinawa National College of Technology, 905 Aza-Henoko, Nago, Okinawa 905-2191, Japan

## Abstract

In order to explore marine natural resources using remote robotic sensor or to enable rapid information exchange between ROV (remotely operated vehicles), AUV (autonomous underwater vehicle), divers, and ships, ultrasonic underwater communication systems are used. However, if the communication system is applied to rich living creature marine environment such as shallow sea, it suffers from generated Impulsive Noise so-called Shrimp Noise, which is randomly generated in time domain and seriously degrades communication performance in underwater acoustic network. With the purpose of supporting high performance underwater communication, a robust digital communication method for Impulsive Noise environments is necessary. In this paper, we propose OFDM ultrasonic communication system with diversity receiver. The main feature of the receiver is a newly proposed Frequency Domain Diversity Combined Impulsive Noise Canceller. The OFDM receiver utilizes 20–28 KHz ultrasonic channel and subcarrier spacing of 46.875 Hz (MODE3) and 93.750 Hz (MODE2) OFDM modulations. In addition, the paper shows Impulsive Noise distribution data measured at a fishing port in Okinawa and at a barge in Shizuoka prefectures and then proposed diversity OFDM transceivers architecture and experimental results are described. By the proposed Impulsive Noise Canceller, frame bit error rate has been decreased by 20–30%.

## 1. Introduction

**Figure 1:**
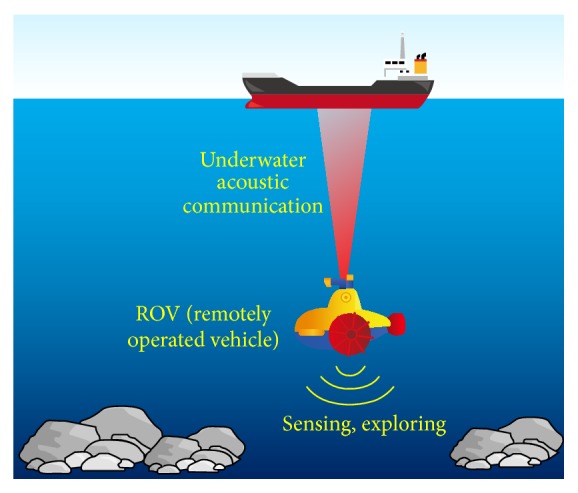
Application of underwater networking.

Underwater wireless communication system [1] will offer a wide variety of applications such as natural disaster warning, remote control of offshore objects, and discovery of new resources at the bottom of ocean. Emerging underwater wireless network devices can be equipped onto underwater vehicles or robots with sensors and video cameras. Then system in ships can access those sensors and video cameras through underwater wireless network by acoustic wireless link.  Figure 1 shows an example to control a bottom sea ROV (remotely operated vehicles), which engages exploring natural resources by sensors, from water surface operator in ship through underwater network. Since many living creatures in marine usually attach to communication devices and they generate impulse type acoustic noise as an interference, a communication system for underwater application has to be robust for this Impulsive Noise. Then high bandwidth acoustic communication system, which is robust for living creature generating Impulsive Noise, so-called Shrimp Noise, is required.

In this paper, we have designed underwater acoustic Orthogonal Frequency Division Multiplexing (OFDM) communication system with four receiving transducers diversity [2–4]. The proposed system utilizes one TX (transmitting) transducer and four RX (receiving) transducers with center frequency of 24 KHz, 8 KHz bandwidth ultrasonic sound. 161 subcarriers in MODE3 and 81 subcarriers in MODE2 are multiplexes in 8 KHz bandwidth. In order to increase the robustness of the receiver under Impulsive and White Gaussian Noise circumstances, MRC (maximum ration combiner) Frequency Domain Diversity Combined Impulsive Noise Canceller is newly proposed.  Section 2 describes the statistics analysis of the marine creatures' Impulsive Noise based on measurements at a fishing port in Okinawa and at a barge in Shizuoka Prefecture.  Section 3 shows the communication system design details including the proposed Frequency Domain Diversity Combined Impulsive Noise Canceller. Then, experimental results will be disclosed in Section 4. Finally, conclusion is given in Section 5.

## 2. Analysis of Impulsive Noise

**Figure 2:**
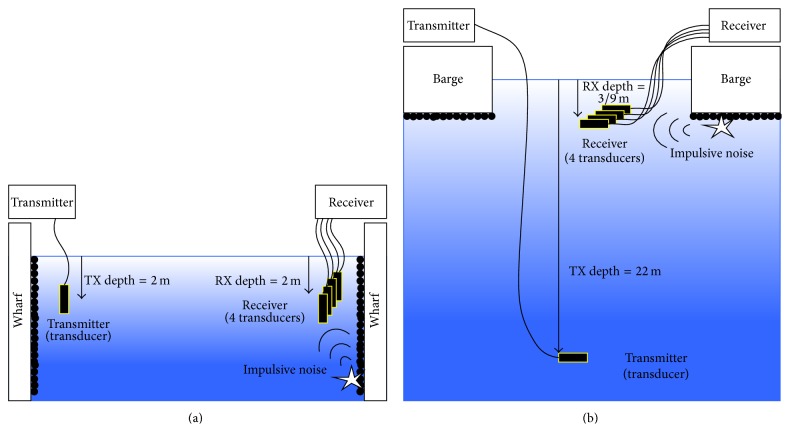
(a) Okinawa experiment at OJIMA fishing port. (b) Shizuoka experiment in UCHIURA barge.

We have performed ocean experiments at two sites. One is OJIMA located at the south of Okinawa Island in Okinawa Prefecture in Japan. The other is barge in UCHIURA-cove in Numazu city in Shizuoka, which is roughly 400 m offshore and the sea depth is 30 m.  Figure 2(a) shows a cross-sectional view of OJIMA experiment site. Both TX and RX transducers are set in the depth of 2 m, and the distance between TX and RX is 71.5 m. The sea depth is roughly 5 m. At the wall of the wharf, sea creatures are expected to generate Impulsive Noise as shown in the figure.  Figure 2(b) shows a cross-sectional view of UCHIURA barge experiment site. TX transducer is set in the depth of 22 m, and four RX transducers are set in the depth of 3 m and 9 m. At the bottom of the barge, sea creatures are expected to generate Impulsive Noise as shown in the figure.

**Figure 3:**
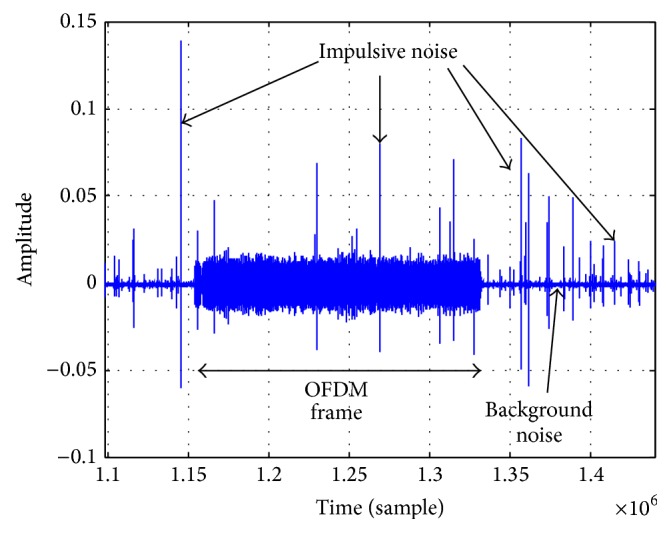
Received signal with many Impulsive Noise Peaks.

**Figure 4:**
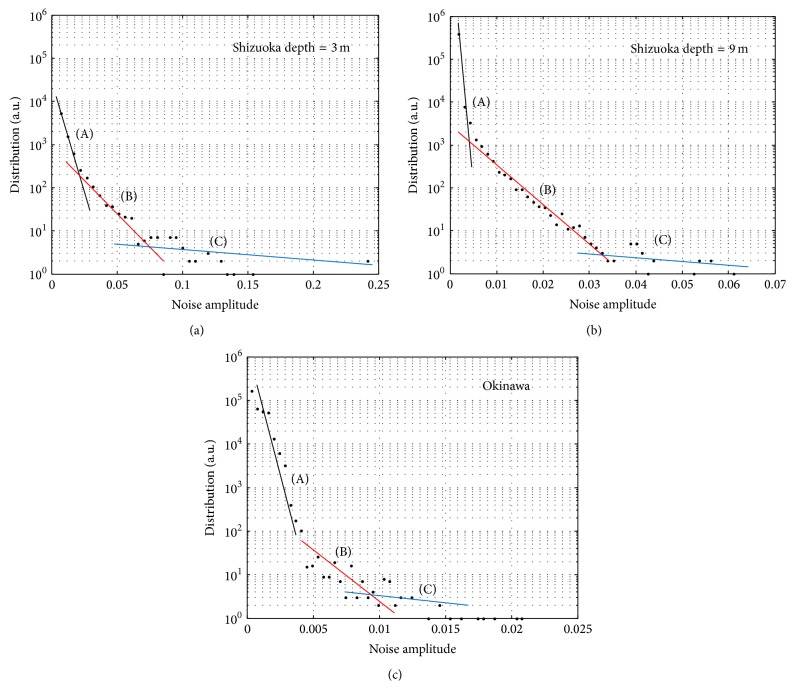
(a) Background noise distribution at Shizuoka (depth = 3 m). (b) Background noise distribution at Shizuoka (depth = 9 m). (c) Background noise distribution at Okinawa.

**Figure 5:**
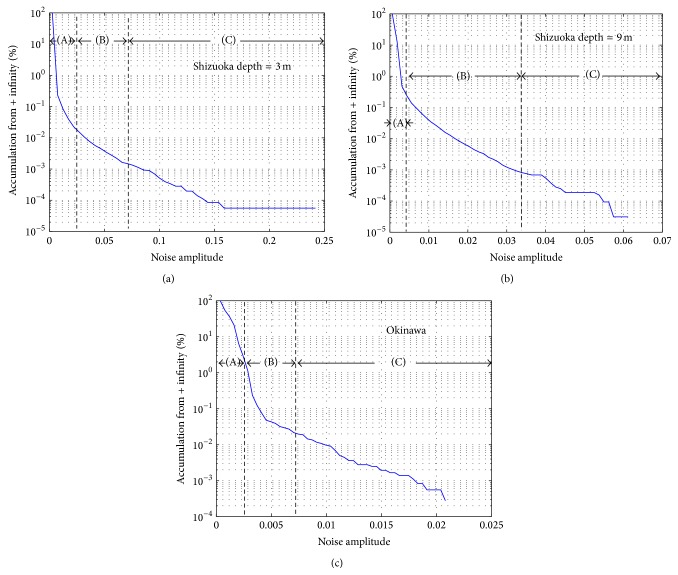
(a) Accumulated background noise from + infinity at Shizuoka (depth = 3 m). (b) Accumulated background noise from + infinity at Shizuoka (depth = 9 m). (c) Accumulated background noise from + infinity at Okinawa.

Figure 3 shows a measured received signal of one RX transducer at UCHIURA barge experiment with RX depth of 3 m. The center portion of the signal is OFDM frame and another portion corresponds to No signal. Many large Impulsive Noises are observed. Obviously, a large impulse gives serious damage to the OFDM signal demodulation. Then the background noise amplitude distributions are analyzed. This analysis only observes the background noise and no OFDM frame signal is included. Figures 4(a) and 4(b) are background noise distribution at Shizuoka with depth = 3 m and 9 m, respectively. Figures 5(a) and 5(b) are accumulated background noise from + infinity at Shizuoka site with depth = 3 m, 9 m. From the figures, more than 99% of the signals correspond to (a) area, which has relatively small signal amplitude, that is, low noise power. However, as shown in figures, relatively large signal amplitude (large noise power) is observed in the areas (b) and (c). Comparing Figures 4(a) with 4(b), shallow depth of 3 m shows larger signal amplitude. Therefore, the source of the Impulsive Noise can be expected at the bottom of the barge.

Background noise distribution data at OJIMA site is also shown in Figure 4(c). Although the OJIMA site which locates in semitropical area is a very much different condition as shown in Figure 2(a) comparing with the Shizuoka barge 2(b), similar distribution data is obtained.  Figure 5(c) is accumulated background noise from + infinity at OJIMA site. From the figure, roughly 98% of the signals correspond to (a) area, which has relatively small signal amplitude, that is, low noise power. Therefore, in order to achieve reliable digital communication in marine environment, Impulsive Noise mitigation is required. In the following section, detail of proposed OFDM receiver with Frequency Domain Diversity Combined Impulsive Noise Canceller will be described.

## 3. Communication System Design with Impulsive Noise Canceller

### 3.1. Communication System Architecture [5, 6]

**Figure 6:**
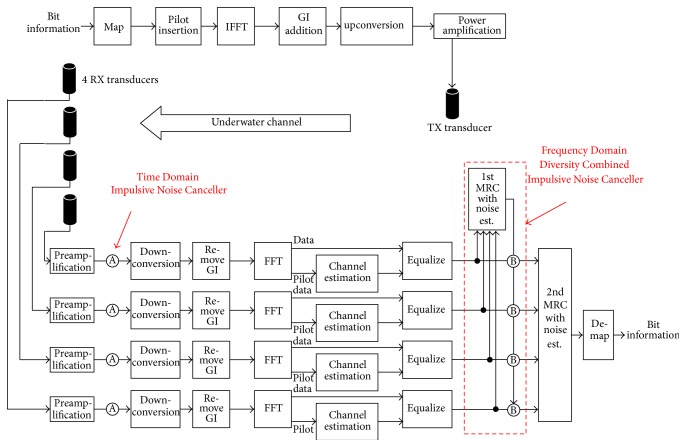
Block diagram of OFDM communication system with Frequency Domain Diversity Combined Impulsive Noise Canceller.

Figure 6 shows a block diagram of OFDM communication system with Frequency Domain Diversity Combined Impulsive Noise Canceller. The upper side is a transmitter (TX). Bit information is mapped to QPSK, 16QAM, or 64QAM constellations. Some BPSK modulated pilot patterns are inserted in the stream in order to enable a channel estimation at receiver side (RX). After that, OFDM modulation is performed through Inverse Fast Fourier Transform (IFFT). Then Guard Interval (GI) is added to each OFDM symbol. By upconversion block, the modulated baseband signal is upconverted to passband such as 20–28 KHz. TX transducer outputs ultrasonic sound OFDM signal into the sea water. Through the underwater acoustic channel, four RX transducers receive the transmission signal. Then the signals are processed in reverse order such as amplifying, downconverting to baseband signal, removing Guard Interval, and FFT. By using the inserted pilot signal, channel estimation is performed. At the equalizer block, FFT output data is equalized. The circle-A and circle-B in the figure correspond to Time Domain and Frequency Domain Impulsive Noise Cancellation signal processing block.

Following the equalizer, there are two stages of 4 inputs Maximum Ratio Combiner (MRC) blocks. Equation (1) shows the MRC computation [7–9]. MRC(*k*) is the output of each MRC block:

(1) $$\begin{array}{c} MRC\left(\;k\;\right)=\frac{{EQ\left(k\right)}_{1}\cdot {\left|{H\left(k\right)}_{1}\right|}^{2}/\sigma_{n1}^{2}{+EQ\left(k\right)}_{2}\cdot {\left|{H\left(k\right)}_{2}\right|}^{2}/\sigma_{n2}^{2}+{EQ\left(k\right)}_{3}\cdot {\left|{H\left(k\right)}_{3}\right|}^{2}/\sigma_{n3}^{2}+{EQ\left(k\right)}_{2}\cdot {\left|{H\left(k\right)}_{4}\right|}^{2}/\sigma_{n4}^{2}}{{\left|{H\left(k\right)}_{1}\right|}^{2}/\sigma_{n1}^{2}+{\left|{H\left(k\right)}_{2}\right|}^{2}/\sigma_{n2}^{2}+{\left|{H\left(k\right)}_{3}\right|}^{2}/\sigma_{n3}^{2}+{\left|{H\left(k\right)}_{4}\right|}^{2}/\sigma_{n4}^{2}}. \end{array}$$

Here, EQ(*k*)_1–4_ are 4 equalizer outputs, *H*(*k*)_1–4_ are Channel Transfer Functions (CTFs) of each underwater channel, index *k* corresponds to subcarrier number, and *σ*_*n*1−4_^2^ are estimated average noise power at each underwater channel. Highlight of the system design is frequency domain Impulsive Noise Canceller with two-stage MRC combiners, although the reference paper [10] shows a frequency domain Impulsive Noise Canceller but no diversity combiners. The 1st MRC combiner's outputs are utilized to suppress Impulsive Noise by frequency domain Impulsive Noise Cancellation signal processing block circle-B.

Table 1 summarizes detail system parameters. Sampling frequency of the system is 96 KHz and two kinds of operation modes are supported such as MODE2 and MODE3, which correspond to 93.750 Hz and 46.875 Hz subcarrier spacing, respectively. 8 KHz of communication bandwidth is divided into 81 subcarriers in MODE2 and 161 subcarriers in MODE3. In order to simplify the design, Guard Interval (GI) length of a half size of OFDM symbol length *T* is used.

### 3.2. Time and Frequency Impulsive Noise Cancelling

**Figure 7:**
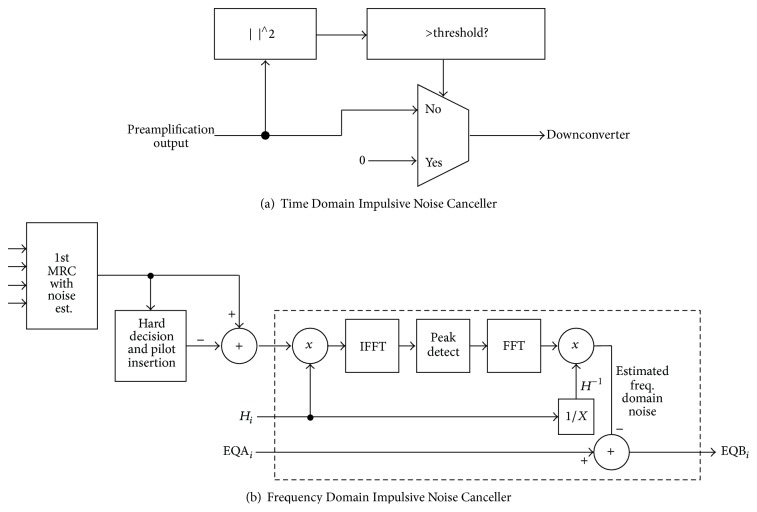
Time and Frequency Domain Impulsive Noise Canceller.

Because of the presence of Impulsive Noise so-called Shrimp Noise, time and frequency domain Impulsive Noise Cancelling (time-IMP and freq.-IMP) methods [4, 5] are employed and further combined with carrier diversity combiner. The block diagram of time-IMP (circle-A in Figure 6) is shown in Figure 7(a). It detects Impulsive Noise samples in time domain and then removes the Impulsive Noise samples according to

(2) $$\begin{array}{c} y\left(\;n\;\right)=\left\{\begin{array}{cc} r\left(n\right) & \text{if }{\left|r\left(n\right)\right|}^{2}\leq \alpha \cdot P_{avg}\\ 0 & \text{otherwise.} \end{array}\right. \end{array}$$

Here *r*(*n*) is *n*th time sample of received signal after preamplifier, *P*_avg_ is the average power of an OFDM symbol, and *α* is threshold parameter. Obviously, if the power of input signal *r*(*n*) exceeds the threshold of  *α* · *P*_avg_, the signal is replaced by zero. This method is called “blanking” [10, 11]. In order to determine the threshold parameter  *α*, cut and try experimentation was applied and finally *α* = 20 is determined. The system performance is not so sensitive to the parameter.

In addition, a frequency domain Impulsive Noise Cancellation is applied. The detail of freq.-IMP (circle-B in Figure 6) is shown in Figure 7(b). The 1st MRC block generates the combined consternation of 4 branches equalizers. Then the following processing is applied in hard decision and pilot insertion block:

1. Subcarriers, which should be silent (no data or pilots), are set to zero.
2. Subcarriers, which are used as pilots, are replaced by known pilot values.
3. Subcarriers, which are used for data transmission, are demapped to nearest position in constellation plot.

Then succeeding subtraction block computes frequency domain noise components. In the following blocks, the frequency domain noise components are converted to a Time Domain Impulsive Noise. The frequency domain noise is multiplied with Channel Transfer Function of branch *i* (*H*_*i*_, *i* = 1 to 4) and is IFFTed. If the output of IFFT has a large peak, it can be considered as estimated Time Domain Impulsive Noise signal *u*(*n*) according to

(3) $$\begin{array}{c} u\left(\;n\;\right)=\left\{\begin{array}{cc} \hat{d}\left(n\right) & \text{if }{\left|\hat{d}\left(n\right)\right|}^{2}\geq \beta \cdot \sigma^{2}\\ 0 & \text{otherwise.} \end{array}\right. \end{array}$$

Here $\hat{d}\left(n\right)$ is *n*th time sample of IFFT output, *σ* ^2^ is the average power of the IFFT output, and *β* is threshold parameter. Obviously, if the power of input signal $\hat{d}(n)$ exceeds the threshold of  *β* · *σ* ^2^, the signal is considered as an Impulsive Noise. Then the estimated Time Domain Impulsive Noise signal *u*(*n*) is converted to frequency domain by FFT block and division by *H*_*i*_. Finally, the estimated frequency domain Impulsive Noise is withdrawn from equalized signal of branch *i* (*i* = 1 to 4) EQA_*i*_. Then the noise reduced outputs EQB_*i*_ (*i* = 1 to 4) are obtained and then they are combined in the 2nd MRC block to generate further noise reduced constellation. The parameter *β* is determined by cut and try experimentations and *β* = 8 is finally applied to the system. The obtained noise cancel performance is not so sensitive to the parameter *β*.

## 4. Experimental Results

**Figure 8:**
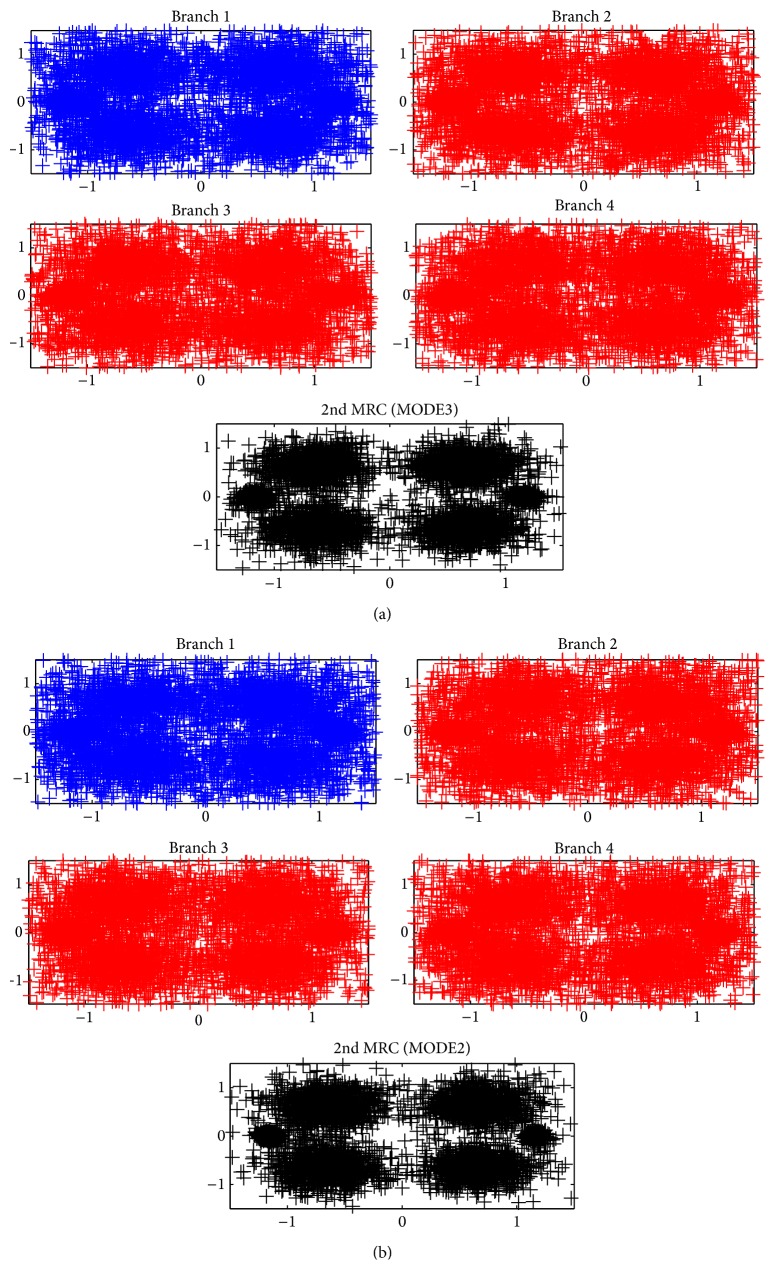
(a) MODE3 QPSK constellations (branches 1 to 4 and MRC, Okinawa). (b) MODE2 QPSK constellations (branches 1 to 4 and MRC, Okinawa).

Table 2 summarizes ocean experiment parameters for both Okinawa OJIMA fishing port site and Shizuoka UCHIURA barge site. In Okinawa site, horizontal underwater communication is carried out while vertical communication is experimented in Shizuoka site, for The four RX transducers are placed as linear array with 20 cm pitch. Figures 8(a) and 8(b) are MODE3 and MODE2 constellations in Okinawa site, respectively. Upper four constellations correspond to branches 1 to 4 2nd MRC inputs. The bottom constellation is 2nd MRC output. The horizontal axis of each figure shows real part of digital modulation, while the vertical axis corresponds to imaginary part. According to the figures, carrier diversity combining MRC successfully reduces variance of constellation points.

**Figure 9:**
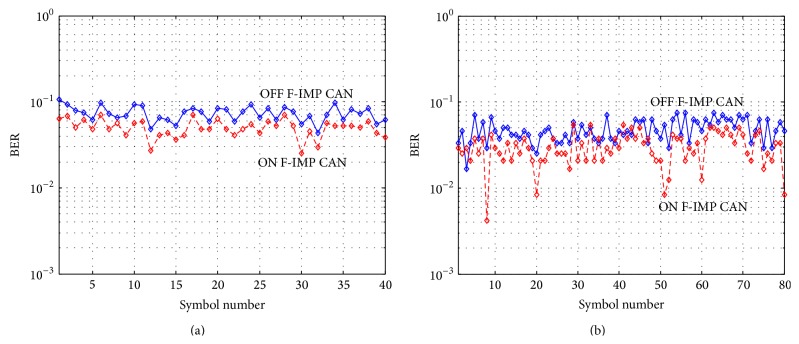
(a) MODE3 BER comparison for 16QAM at Okinawa. (b) MODE2 BER comparison for 16QAM at Shizuoka.

Figures 9(a) and 9(b) are the OFDM symbol by symbol bit error rate (BER) comparisons between frequency domain Impulsive Noise cancellation ON and OFF for MODE3 16QAM modulation in Okinawa site and MODE2 16QAM in Shizuoka site, respectively. The solid line corresponds to freq.-IMP OFF and the dashed line corresponds to ON. Horizontal axis shows BER and vertical axis shows OFDM symbol number. According to the figures, the proposed Frequency Domain Diversity Combined Impulsive Noise Canceller effectively reduces symbol by symbol BER values.

**Figure 10:**
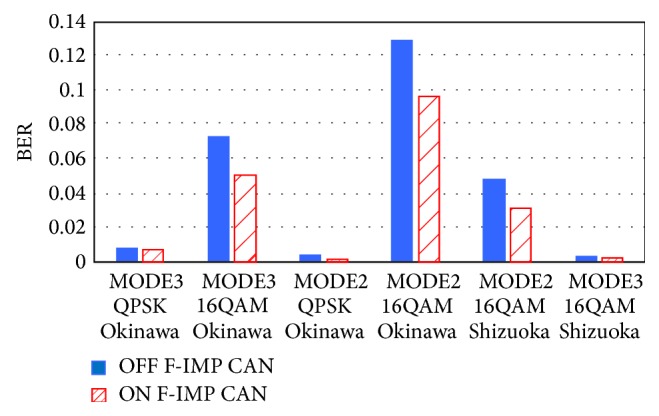
Summary of BER comparison for Frequency Domain. Diversity Combined Impulsive Noise Canceller.

Figure 10 summarizes BER comparisons for Frequency Domain Diversity Combined Impulsive Noise Canceller for freq.-IMP ON and OFF. In spite of place of experiment site, approximately 20–30% of BER reduction has been obtained by the proposed Frequency Domain Diversity Combined Impulsive Noise Canceller.

**Figure 11:**
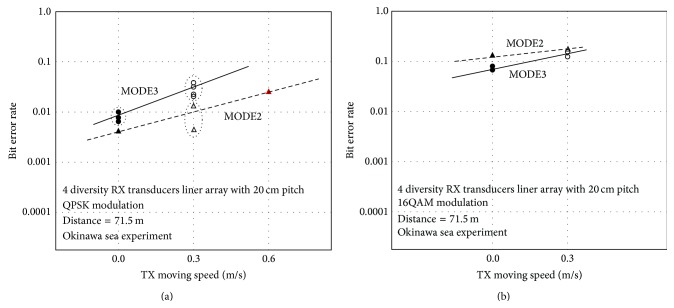
(a) Communication system performance using QPSK modulation. (b) Communication system performance using 16QAM modulation.

Finally, Figures 11(a) and 11(b) show measured communication system performance in Okinawa site with enabling the proposed Impulsive Noise Canceller. In addition, BER data is also measured with moving the TX transducer speed such as 0.0 m/s (stable), 0.3, and 0.6 m/s for QPSK modulation and 0.0 m/s (stable) and 0.3 m/s for 16QAM. Since MODE2 OFDM symbol length of 10.667 ms as shown in Table 1 is half of MODE3, the Guard Interval length of MODE2 is shorter than MODE3. Therefore it is considered that high level modulation of 16QAM of MODE2 is largely affected by Inter Symbol Interference (ISI). As you can see from those figures, MODE2 shows higher robustness for moving cases such as strangeness for Doppler Effect because of shorter OFDM symbol length of MODE2. Once forward error correction such as TURBO CODE with code rate *R* = 1/3 is applied to this system, all error free communications (BER = 0) for QPSK modulation of all speeds and 16QAM stable case have been confirmed.

## 5. Conclusion

In this paper, an ultrasonic OFDM transceiver architecture with four diverse receivers supporting 2 MODEs is proposed with enhancement of Impulsive Noise insusceptibility. It utilizes 20–28 KHz ultrasonic channel and subcarrier spacing of 46.875 Hz (MODE3) and 93.750 Hz (MODE2) OFDM modulations.

Two different environment ocean experiments were conducted to evaluate the statistics of Impulsive Noise so-called Shrimp Noise, which is generated by living creatures in marine environment. To deal with challenges posed by the Impulsive Noise interference, we have employed a Frequency Domain Diversity Combined Impulsive Noise Canceller.

Measured data at a fishing port in Okinawa and at a barge in Shizuoka Prefecture are disclosed and in both environment randomly generated Time Domain Impulse Noise distribution is observed. Although more than 90 to 99% of background noise shows AWGN type distribution, less than 1 to 10% noise components show large amplitude such as Impulsive Noise.

By enabling the proposed MRC combined Frequency domain Impulsive Noise Canceller, 20–30% BER reduction has been successfully obtained. In addition, 71.5 m shallow fishing port horizontal underwater communication BER is also shown using QPSK and 16QAM modulations. By applying TURBO *R* = 1/3 forward error correction, all error free communications using QPSK and 16QAM with not moving case are also confirmed.

## Figures and Tables

**Table 1:** System parameters.

Parameters	Mode	
	2	3
TX-RX elements	1 TX and 4 RX transducer	
Sampling frequency	96000 Hz	
TX center frequency	24000 Hz	
Band width	8000 Hz	
FFT size	1024	2048
OFDM symbol length *T*	10.667 ms	21.333 ms
GI length	0.5*T*	0.5*T*
Subcarrier spacing	93.75 Hz	46.875 Hz
Number of subcarriers	81	161

**Table 2:** Ocean experiment parameters.

Parameters	Place	
	Okinawa	Shizuoka
Type of experiment site	Fishing port	Barge
Number of transducers	1 TX and 4 RX	1 TX and 4 RX
RX transducer array	Linear with 20 cm pitch	Linear with 20 cm pitch
Modulation	QPSK/16QAM	QPSK/16QAM
TX-RX distance	71.5 m	13 m
Transmission direction	Horizontal	Vertical
Ocean depth	5 m	30 m
Transducer depth (TX/RX)	2 m/2 m	22 m/9 m
